# Changes in terpene biosynthesis and submergence tolerance in cotton

**DOI:** 10.1186/s12870-023-04334-4

**Published:** 2023-06-21

**Authors:** Liangqing Sun, Junjuan Wang, Yupeng Cui, Ruifeng Cui, Ruiqing Kang, Yuexin Zhang, Shuai Wang, Lanjie Zhao, Delong Wang, Xuke Lu, Yapeng Fan, Mingge Han, Chao Chen, Xiugui Chen, Lixue Guo, Wuwei Ye

**Affiliations:** 1 Institute of Cotton Research of Chinese Academy of Agricultural Sciences/Research Base, Anyang Institute of Technology, State Key Laboratory of Cotton Biology, Anyang, 455000 Henan China; 2 Cotton Research Institute of Jiangxi Province, Jiujiang, 332105 Jiangxi China

**Keywords:** Submergence, Transcriptome, Terpene biosynthesis, Cotton, Respiratory metabolism, Cotton

## Abstract

**Background:**

Flooding is among the most severe abiotic stresses in plant growth and development. The mechanism of submergence tolerance of cotton in response to submergence stress is unknown.

**Results:**

The transcriptome results showed that a total of 6,893 differentially expressed genes (DEGs) were discovered under submergence stress. Gene Ontology (GO) enrichment analysis showed that DEGs were involved in various stress or stimulus responses. Kyoto Encyclopedia of Genes and Genomes (KEGG) pathway analysis indicated that DEGs related to plant hormone signal transduction, starch and sucrose metabolism, glycolysis and the biosynthesis of secondary metabolites were regulated by submergence stress. Eight DEGs related to ethylene signaling and 3 ethylene synthesis genes were identified in the hormone signal transduction. For respiratory metabolism, alcohol dehydrogenase (*ADH*, *GH_A02G0728*) and pyruvate decarboxylase (*PDC*, *GH_D09G1778*) were significantly upregulated but 6-phosphofructokinase (*PFK*, *GH_D05G0280*), phosphoglycerate kinase (*PGK*, *GH_A01G0945* and *GH_D01G0967*) and sucrose synthase genes (*SUS*, *GH_A06G0873* and *GH_D06G0851*) were significantly downregulated in the submergence treatment. Terpene biosynthetic pathway-related genes in the secondary metabolites were regulated in submergence stress.

**Conclusions:**

Regulation of terpene biosynthesis by respiratory metabolism may play a role in enhancing the tolerance of cotton to submergence under flooding. Our findings showed that the mevalonate pathway, which occurs in the cytoplasm of the terpenoid backbone biosynthesis pathway (ko00900), may be the main response to submergence stress.

**Supplementary Information:**

The online version contains supplementary material available at 10.1186/s12870-023-04334-4.

## Background

Flooding is among the most severe abiotic stresses that occur during plant growth and development [1]. Flooding is a general term referring to excessively wet conditions, that is where excess water replaces gas-spaces surrounding roots and/or shoots. It mainly includes four aspects: (1) Waterlogging or soil flooding: only the root-zone is flooded, (2) Partial waterlogging or soil flooding: partial flooding of the root-zone, (3) Submergence refers to the entire plant being underwater (4) Partial submergence: the entire root system and part of above-ground organs are under water [2]. Flooding directly affects the diffusion of oxygen in plant tissues/soil, resulting in hypoxia. Hypoxia greatly disrupts respiration and photosynthesis, which leads to a reduction in the ATP supply and has deleterious effects on normal life activities of plants [3]. When plants are completely submerged, they are deprived of oxygen. Most plant species cannot survive prolonged submergence, but they can temporarily adapt to submergence stress through the Low Oxygen Quiescence Syndrome (LOQS) or the Low Oxygen Escape Syndrome (LOES) [4–6]. Heavy rainfall and flood disasters have become frequent in recent years, and there is an urgent need to study the submergence tolerance of plants and their mechanisms to maintain the effective adaptation of plants to climate change [7, 8].

Reactive oxygen species (ROS) are a normal product of plant cell metabolism. Reactive oxygen can be used as a signal molecule to respond to stress, and excessive ROS is harmful to plant cells. Under prolonged hypoxia condition, excessive ROS can be accumulated, causing membrane lipid peroxidation and altering the structure of proteins and nucleic acids [9]. Malondialdehyde (MDA) is one of the most important products of membrane lipid peroxidation and constitutes a common parameter of membrane damage. Plants have evolved a complex set of enzymatic and non-enzymatic detoxification mechanisms to eliminate oxidative damage caused by ROS [10]. Anti-oxidative enzymes of plants include peroxidase (POD), superoxide dismutase (SOD), etc. Non-enzymatic antioxidants mainly contain a series of antioxidants, such as ASA-glutathione (GSH), ascorbic acid (ASA), lycopene, carotene, and other secondary metabolites [10]. Many studies have reported that secondary metabolites such as GSH, ASA and lycopene enhance plant resistance by reducing ROS [11–14].

Glycolysis is a common starting pathway of aerobic and anaerobic respiration in plants and is a process in which plants anaerobically decompose glucose into pyruvate under the action of a series of enzymes as energy is released [15, 16]. Some intermediate products of glycolysis are important raw materials for the synthesis of secondary metabolites, and the final product, pyruvate, is very biochemically active. Under normoxia, pyruvate is completely oxidized to produce ATP through the TCA cycle. Under hypoxia, pyruvate can produce energy in plants through two alternative pathways. Pyruvate can be converted into lactic acid by *lactate dehydrogenase (LDH)* or gradually converted into ethanol by *pyruvate decarboxylase (PDC)* and *alcohol dehydrogenase* (*ADH)* [17]. There are many previous studies on the energy supply of plants in response to waterlogging or partial stress [18–22]. For example, soybean that was genetically modified with *GmADH2* had an enhanced seed germination ability under waterlogging [19]. The *PDC* activity and resistance to hypoxia are significantly enhanced in *Arabidopsis* by overexpression of *LDH* [23]. Under submerged conditions, plant submergence tolerance is related to carbohydrate accumulation and consumption [5, 22, 24, 25]. However, it is unclear how cotton would cope with energy shortages under complete submergence.

Secondary metabolites can be divided into the following categories according to their chemical structure and properties: terpene-, phenol- and nitrogen-containing secondary compounds [26]. The terpenoid biosynthetic pathway is one of the main metabolic pathways in organisms. Terpenoids produced by this pathway are very large, and more than 30,000 species have been identified [27]. There are two synthetic pathways: the mevalonate pathway (the MVA pathway) in the cytoplasm and pyruvate/glyceraldehyde-3-phosphate pathway (the DXP pathway) in plastids. However, which pathway plays the main role in the response of plants to submergence stress has not been reported so far. Many terpenoids have good antioxidant properties and are the main effective ingredients (antitumor paclitaxel and antimalarial artemisinin, for example) of natural botanicals. Ginsenoside Re isolated and extracted from American ginseng has an antioxidant effect, which can remove internal and external oxidants of cardiomyocytes and protect them from oxidative damage [28]. Studies on the improvement of plant submergence tolerance by scavenging ROS by terpenoids under submergence stress have not been reported.

Cotton is a crop that is sensitive to submergence stress. However, most of recent studies have focused on the cotton growth and yield loss in response to waterlogging [29, 30]. Previous studies on the mechanism of submergence tolerance have been limited to rice, while the mechanism of submergence stress tolerance is unknown in cotton. In this study, the potential mechanism of cotton in response to submergence stress was investigated through transcriptome, physiological and biochemical analyses to provide guidance for cotton breeding and production.

## Results

### Cotton morphological and cytological changes

**Fig. 1:**
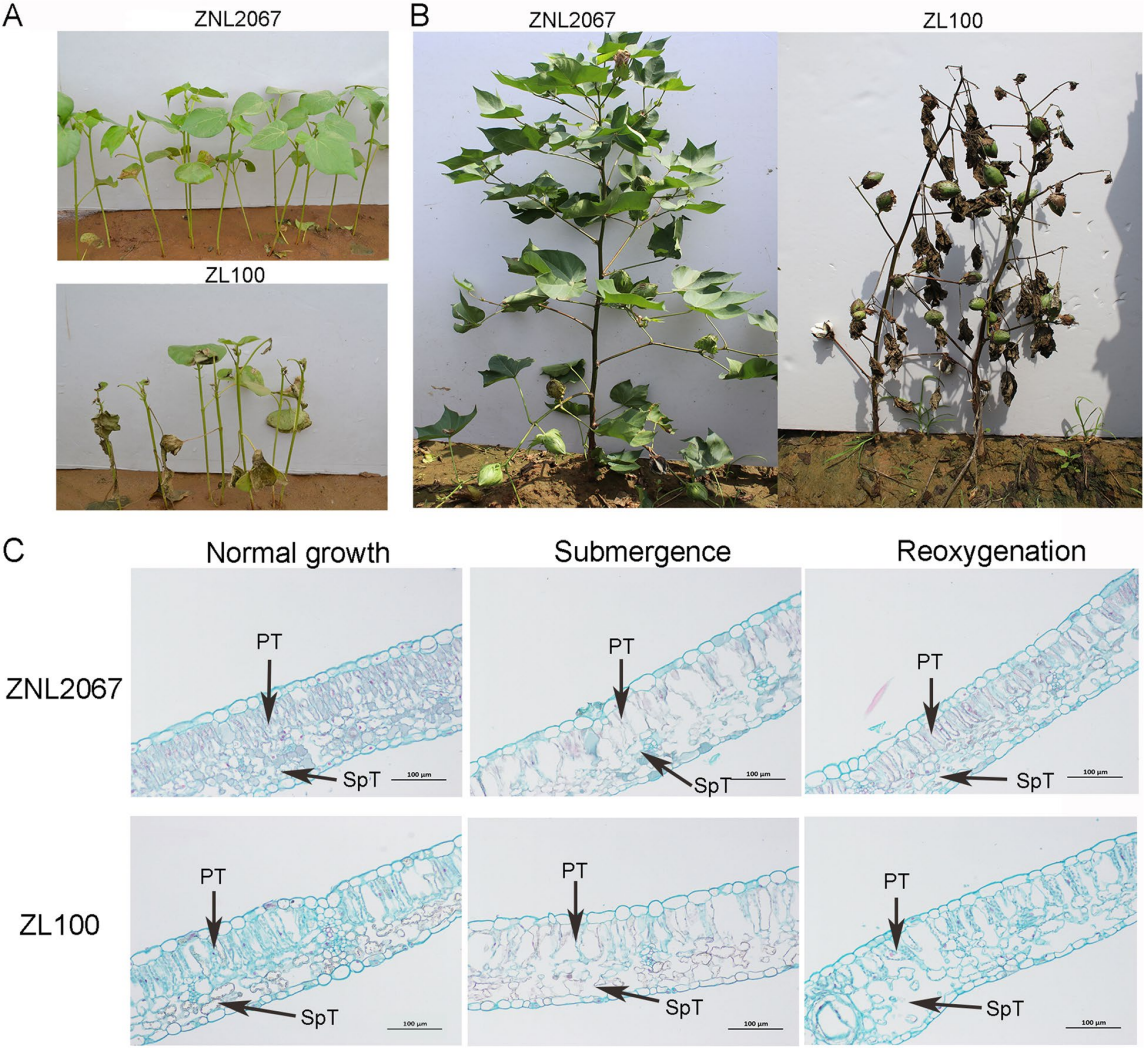
Analysis of the morphological and cytological characteristics of ZNL2067 and ZL100. **A** The field phenotype of ZNL2067 and ZL100 after 3 days of submergence in the three-leaf period; **B** The field phenotype of ZNL2067 and ZL100 after 7 days of submergence in the flowering and boll stage; **C** The leaf cytological characteristics of ZNL2067 and ZL100 at the three-leaf stage with normal growth, submergence stress, and recovery growth after submergence; PT: Palisade tissue; SpT: Spongy tissue

The morphology of ZNL2067 and ZL100 was significantly different at the three-leaf stage after being submerged for 3 days (Fig. 1A). ZNL2067 had no morphological changes and displayed upright stems and green leaves. However, ZL100 showed a severely damaged phenotype with withered leaves, and only young leaves remained partially green. Compared to ZL100, ZNL2067 was more tolerant to submergence stress. The morphological characteristics of ZNL2067 and ZL100 submerged for 7 days also verified this result at the flowering and boll stages (Fig. 1B). Therefore, we chose ZNL2067 as a submergence-tolerant plant material to reveal the underlying mechanism of submergence stress.

To explore the cytological changes in cotton to submergence stress, we observed the cytological characteristics of cotton leaves under three treatments: normal growth, submergence stress, and reoxygenation after submergence. Under submergence stress, the palisade and spongy tissues of ZNL2067 and ZL100 became looser to facilitate oxygen exchange (Fig. 1C). Under reoxygenation after submergence, the palisade and spongy tissues of submergence-tolerant ZNL2067 became tighter (Table 1). At the same time, ZNL2067 was treated with short-term flooding and long-term submergence, and we found that ZNL2067 had advantageous roots (ARs) (Fig. S1).

**Table 1:** Changes of leaf tissue in ZNL2067 and ZL100

Treatment	Palisade tissue thickness (μm)	Sponge tissue Thickness (μm)	leaf thickness	RPS (%)	CTR (%)	SR (%)
2067-Nor	61.34 ± 11.16b	59.79 ± 0.80a	157.32 ± 10.12b	1.025 ± 0.18b	0.39 ± 0.01b	0.38 ± 0.03a
2067-Sub	84.35 ± 14.60a	66.85 ± 4.00b	186.70 ± 14.53a	1.265 ± 0.24b	0.45 ± 0.05a	0.36 ± 0.03a
2067-Reo	55.92 ± 2.37a	29.08 ± 3.88c	113.32 ± 5.63c	1.949 ± 0.26a	0.49 ± 0.02a	0.26 ± 0.02b
100-Nor	80.02 ± 0.97a	55.74 ± 2.37b	176.57 ± 3.76b	1.437 ± 0.05a	0.45 ± 0.01a	0.32 ± 0.01c
100-Sub	78.32 ± 1.96a	64.49 ± 4.89a	182.83 ± 4.10a	1.220 ± 0.10b	0.43 ± 0.01ab	0.35 ± 0.02b
100-Reo	56.74 ± 7.44b	55.96 ± 3.91b	142.32 ± 3.70c	1.023 ± 0.18c	0.40 ± 0.04c	0.39 ± 0.03a

Ratio of palisade tissue/sponge tissue(RPS) = (palisade tissue/sponge tissue) × 100%; Cell tense ratio(CTR) = (Palisade tissue thickness/leaf thickness) × 100%; SR = (Sponge tissue thickness/leaf thickness) × 100%. The significance test was performed using Duncan's method

The difference of lowercase letters after the data indicates a significant difference

### Changes of physiological indicators and ROS in cotton

**Fig. 2:**
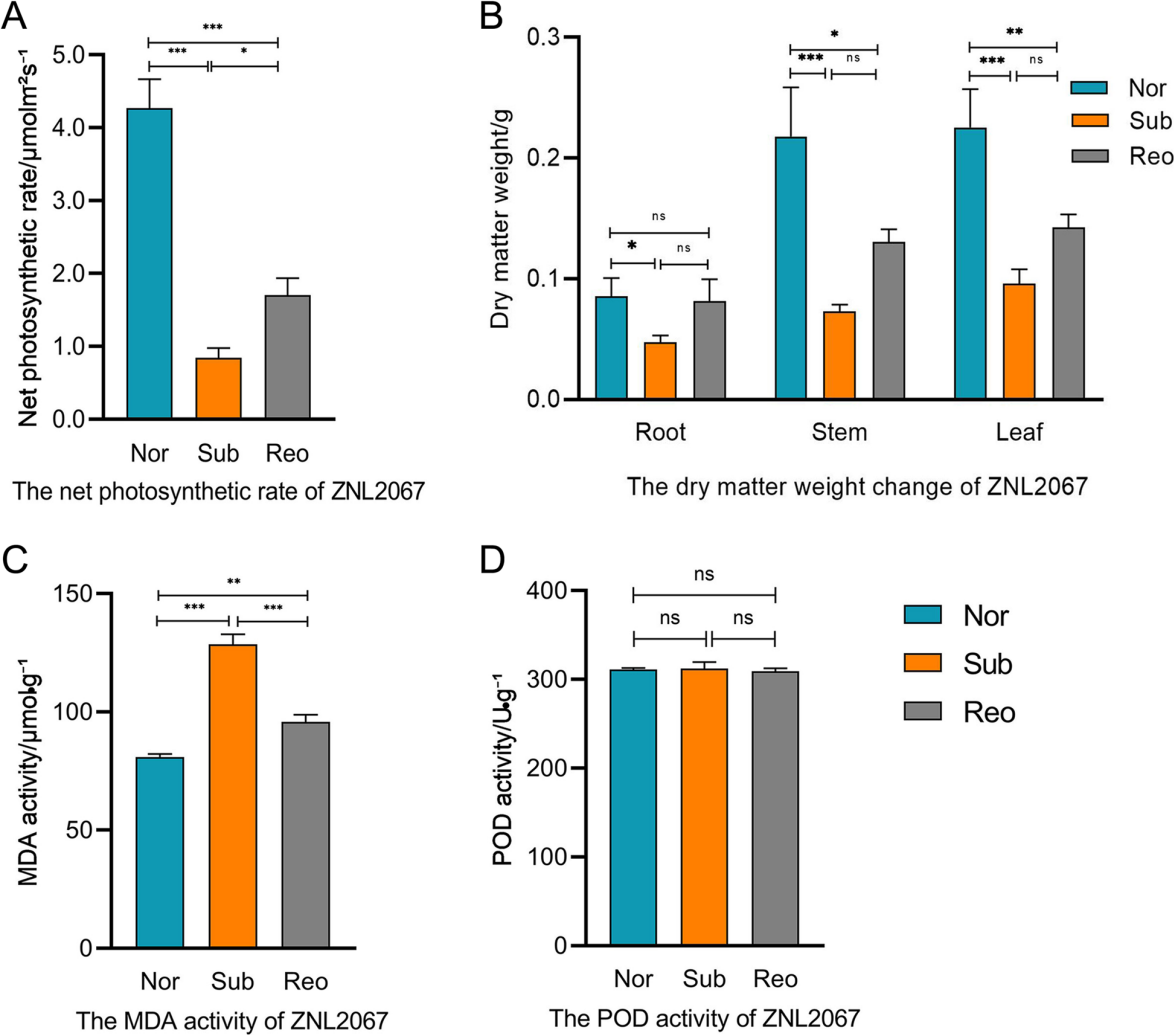
Changes in physiological indicators before and after submergence in ZNL2067. **A** The net photosynthetic rate change of ZNL2067. **B** The dry matter weight change in ZNL2067. **C** The MDA activity of ZNL2067. **D** The POD activity of ZNL2067. Data shown are the mean ± SD (*n* = 3). Nor: normal growth; Sub: submergence; Reo: reoxygenation after submergence. The significance test was performed using Student's t-test. *: *P* < 0.05, **: *P* < 0.01 and ***: *P* < 0.001. Error bars are the standard deviation (SD) of three biological replicates in each treatment group

To study the effects of submergence stress on physiological indicators such as photosynthesis, biological yield of submergence-resistant cotton, we measured the changes in net photosynthetic rate and dry matter. The net photosynthetic rate of ZNL2067 was significantly lower than that under normal growth under submergence treatment. After 3 days of reoxygenation, the net photosynthetic rate of ZNL2067 was significantly higher than that of the submergence treatment (Fig. 2A). The dry matter weights of roots, stems and leaves of ZNL2067 were significantly lower than those of normal growth under submergence treatment. Under reoxygenation after submergence, the dry matter weight of stems and leaves of ZNL2067 increased compared with that of stems and leaves treated with submergence (Fig. 2B). This showed that ZNL2067 resumed growth after reoxygenation.

In order to study the effect of submergence stress on reactive oxygen species (ROS) in submergence-resistant cotton, the changes of MDA and POD were measured. The MDA value increased significantly after 3 days of submergence stress and decreased significantly after reoxygenation (Fig. 2C). Submergence stress promoted the accumulation of ROS, which in turn promoted the aggravation of cotton oxidative damage. The activity of POD was not significantly different between the different treatments (Fig. 2D). We speculated that ZNL2067 may have eliminated excess ROS after being submerged for 3 days. Based on the above physiological and biochemical results, ZNL2067 eliminated the ROS damage and resisted short-term submergence stress.

### Transcriptome sequencing and alignment

**Fig. 3:**
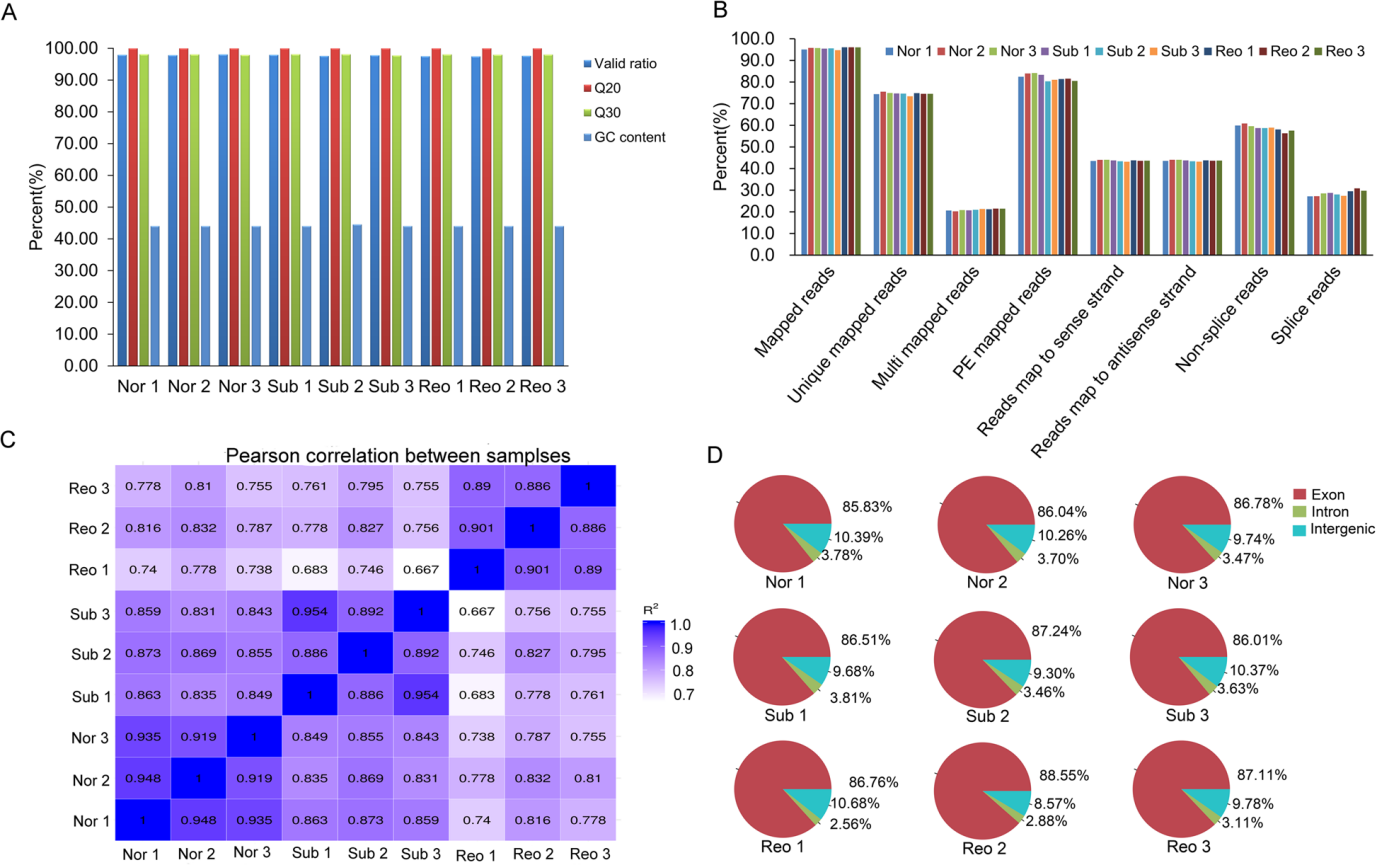
Sequencing quality analysis. **A** Sequencing quality statistics. **B** Reads comparison statistical analysis. **C** Pearson’s correlation coefficient (R^2^) between different samples. **D** Reads comparison region distribution. The colors red, light blue and light green represent exons, introns and intergens, respectively. Nor: normal growth; Sub: submergence; Reo: reoxygenation after submergence

To investigate the molecular response of cotton to submergence stress, we analyzed the RNA-seq data during normal growth (Nor), submergence (Sub) and reoxygenation after submergence (Reo), with three biological replicates. We utilized 3 days of submergence treatment and 3 days of reoxygenation as the sampling times. We obtained 64.27 Mb, 72.63 Mb and 74.45 Mb of average valid reads, containing 6.43 Gb, 7.26 Gb and 7.45 Gb of average valid bases, respectively (Table 2). The valid read ratios of nine libraries were all above 97.40%; the percentages of Q20 and Q30 were above 99.98% and 97.94%, respectively, and the GC content was at least 44.00% (Fig. 3A). The mapped read ratio of each sample to the reference genome TM-1 (http://ibi.zju.edu.cn/cotton/) was more than 95% (Fig. 3B). The *R*^2^ values between samples from the same treatment were all above 0.886 (Fig. 3C). The mapped valid read ratio of each sample to the exon region of the reference genome was at least 85.83% (Fig. 3D). These results showed that the quality of the sequencing data met the experimental requirements.

**Table 2:** Summary of sequence reads after filtering

Samples	Raw read (Mb)	Raw base (Gb)	Valid read (Mb)	Valid base (Gb)	Valid ratio (%)	Q20 (%)	Q30 (%)	GC content (%)
Normal growth	64.64	6.56	64.27	6.43	97.91	99.98	97.98	44.00
Submergence	74.31	7.43	72.63	7.26	97.74	99.98	97.94	44.17
Reoxygenation	76.37	7.64	74.45	7.45	97.48	99.98	97.98	44.00

### Analysis of DEGs

**Fig. 4:**
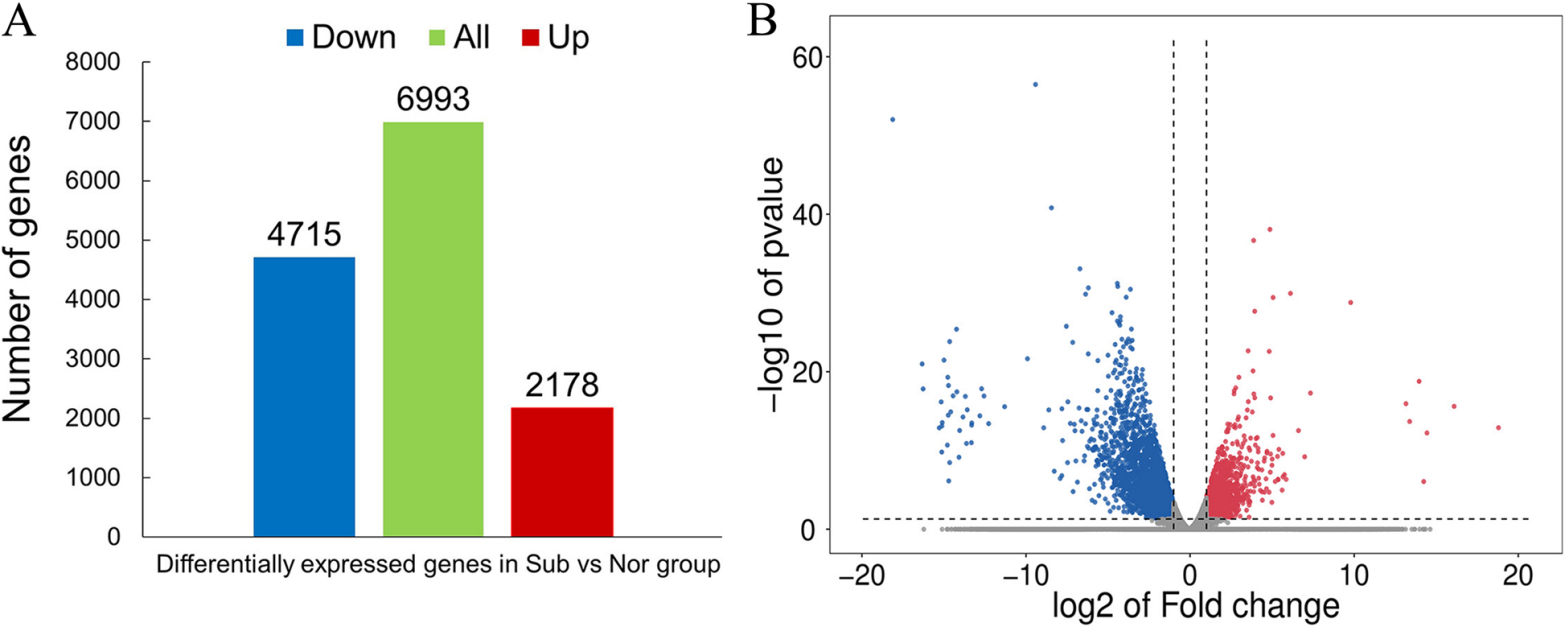
Statistical analysis (**A**) and volcano plot (**B**) of DEGs in response to submergence treatments. Nor: normal growth; Sub: submergence. Red represents the up-regulated genes, blue represents the down-regulated genes, and green represents the total number of DEGs

To identify DEGs of cotton in response to submergence stress, DEGs of the Sub vs. Nor treatment were obtained with a fold change of ≥ 2 and FDR of < 0.01 as the screening criteria, and 6,893 DEGs were identified (Fig. 4). The number of upregulated genes in each treatment was 2,178, and the number of downregulated genes was 4,715, respectively. These DEGs were important candidate genes for further research.

### GO enrichment analysis of DEGs

**Fig. 5:**
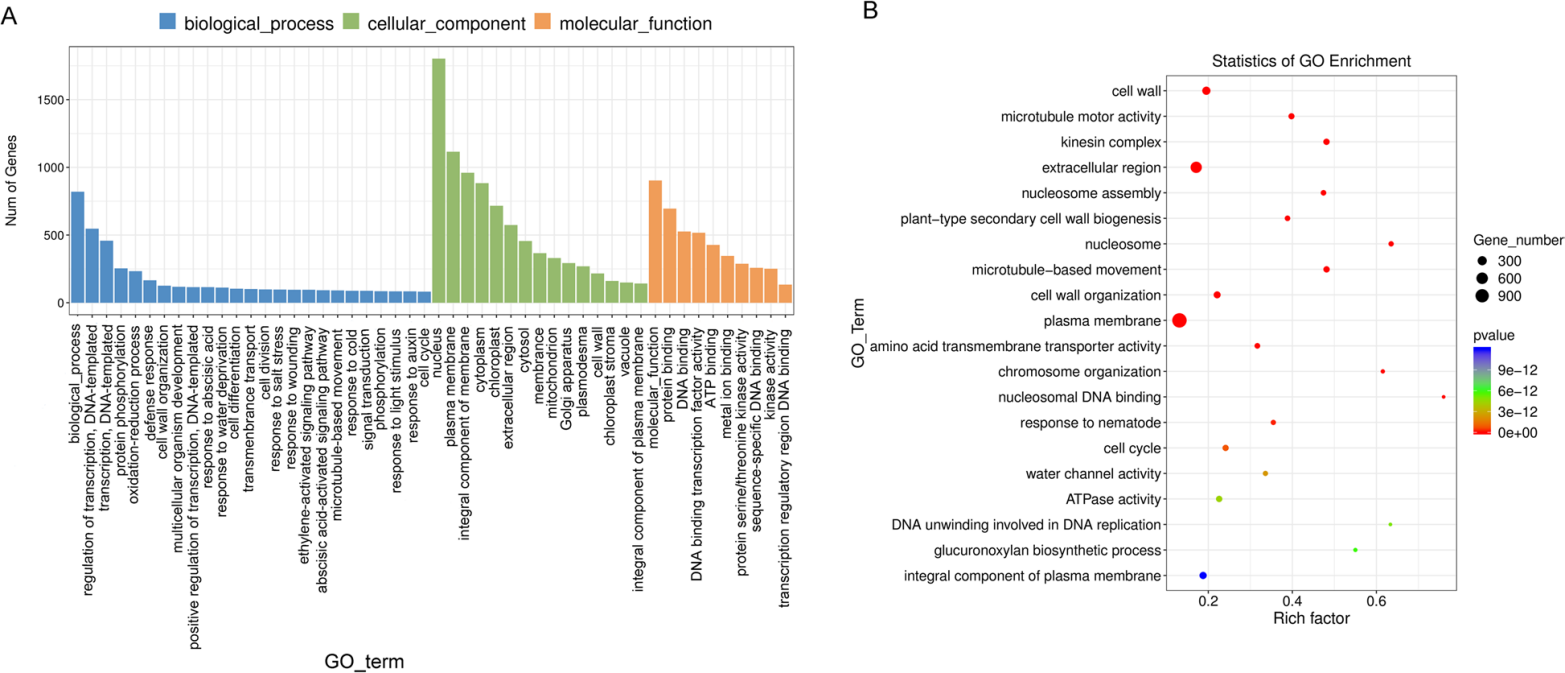
GO term enrichment analysis of DEGs from the Sub vs. Nor group

DEGs of submergence stress were further studied by GO function analysis (Fig. 5). Under submergence stress, the biological processes of DEGs were mainly enriched in protein phosphorylation (GO:0006468), oxidation–reduction process (GO:0055114), regulation of transcription, DNA-templated (GO:0006355), defense response (GO:0006952), cell wall organization (GO:0071555), ethylene-activated signaling pathway (GO:0009873) and cell differentiation (GO:0030154), indicating that cotton responds to submergence stress by regulating protein metabolism, DNA synthesis speed, defense response, and redox reaction. The plasma membrane was the most enriched cell component, indicating that the cotton plasma membrane was very important for the response to submergence stress. The molecular functions of DEGs under reoxygenation were mainly enriched in DNA binding (GO:0003677), protein serine/threonine kinase activity (GO:0004674), sequence-specific DNA binding (GO:0043565), kinase activity (GO:0016301), transferase activity, transferring glycosyl groups (GO:0016757), protein kinase activity (GO:0004672) and ATPase activity (GO:0016887).

**Fig. 6:**
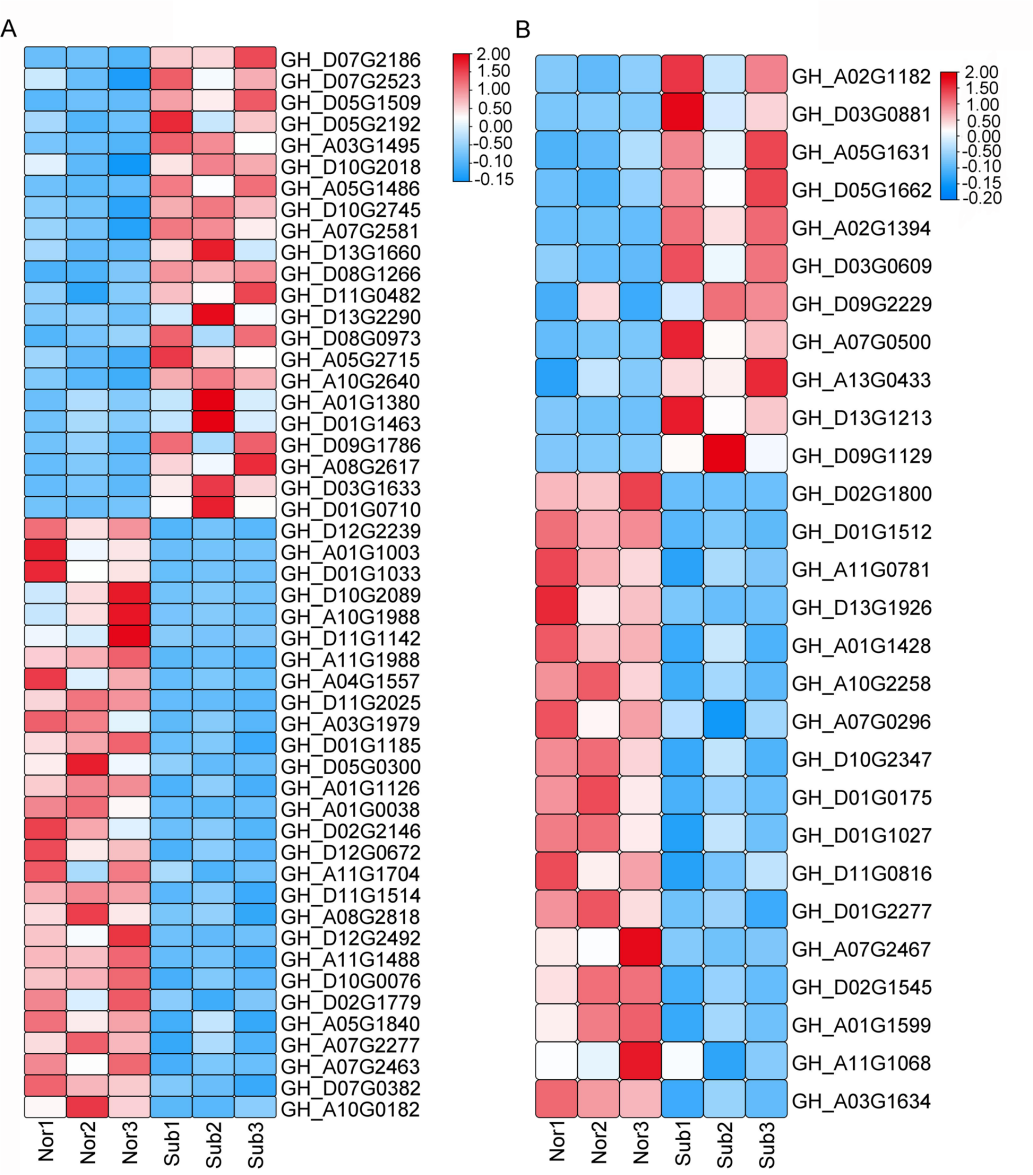
Heatmap of genes associated with the defense response (**A**) and redox (**B**) in submergence treatment. Nor: normal growth; Sub: submergence

We analyzed the redox reaction genes: 106 genes were upregulated and 127 genes were downregulated, and most of them were concentrated in thioredoxin, POD, glyceraldehyde 3-phosphate (GA-3P) dehydrogenase, etc. (Fig. 6A). In defense responses, 134 genes were upregulated and 198 genes were downregulated. Most of the genes were concentrated in ethylene transcription factor, 1-aminocyclopropane-1-carboxylic acid synthase, and E3 ubiquitin protein ligase (Fig. 6B).

### KEGG pathways of DEGs

**Fig. 7:**
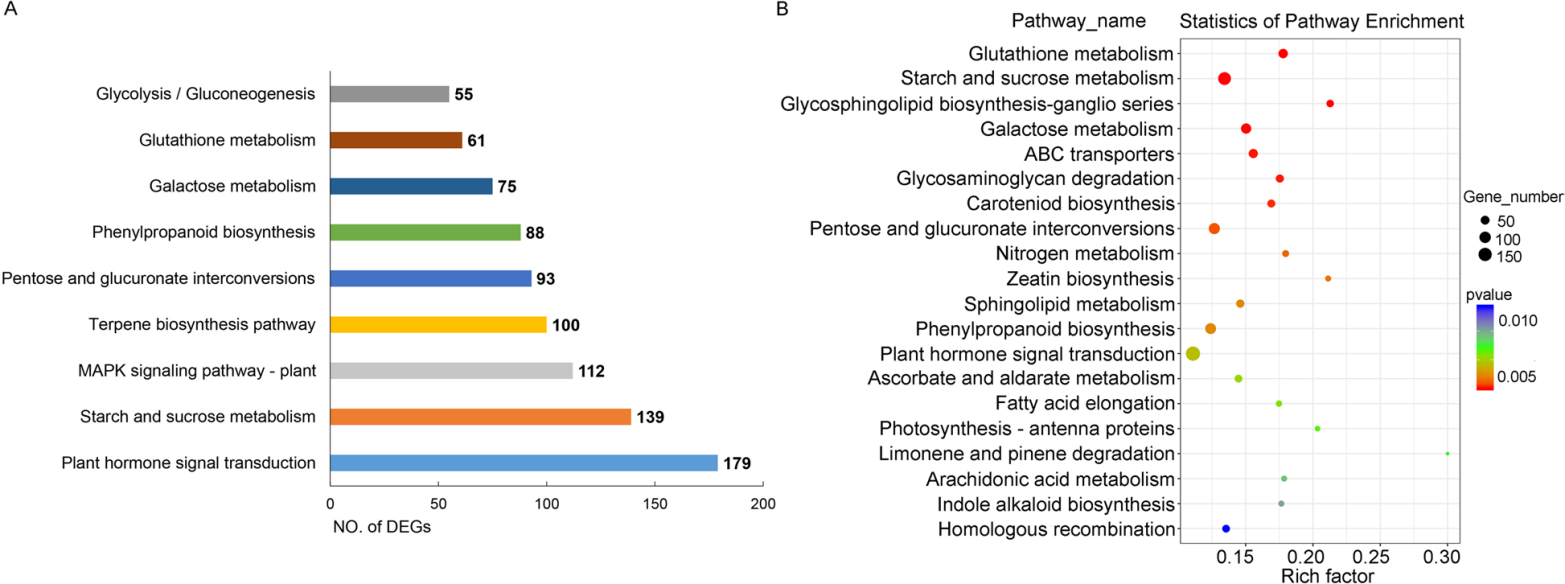
Statistics of DEGs in different KEGG pathways

To determine the main enriched pathways of cotton in response to submergence stress, DEGs under submergence stress were analyzed by KEGG enrichment analysis. In the submergence treatment (Sub vs. Nor), 3203 DEGs were assigned to 131 pathways in the KEGG database. Starch and sucrose metabolism (ko00500), plant hormone signal transduction (ko04075), MAPK signaling pathway (ko04016), phenylpropanoid biosynthesis (ko00940), galactose metabolism (ko00052), glycolysis/gluconeogenesis (ko00010), carotenoid biosynthesis (ko00906), glutathione metabolism (ko00480), and terpene metabolism were significantly enriched terms (Fig. 7). These pathways were related to plant signal transduction, carbohydrate metabolism and secondary metabolism in plants under abiotic stress.

### DEGs analysis of the respiratory metabolism pathway

**Fig. 8:**
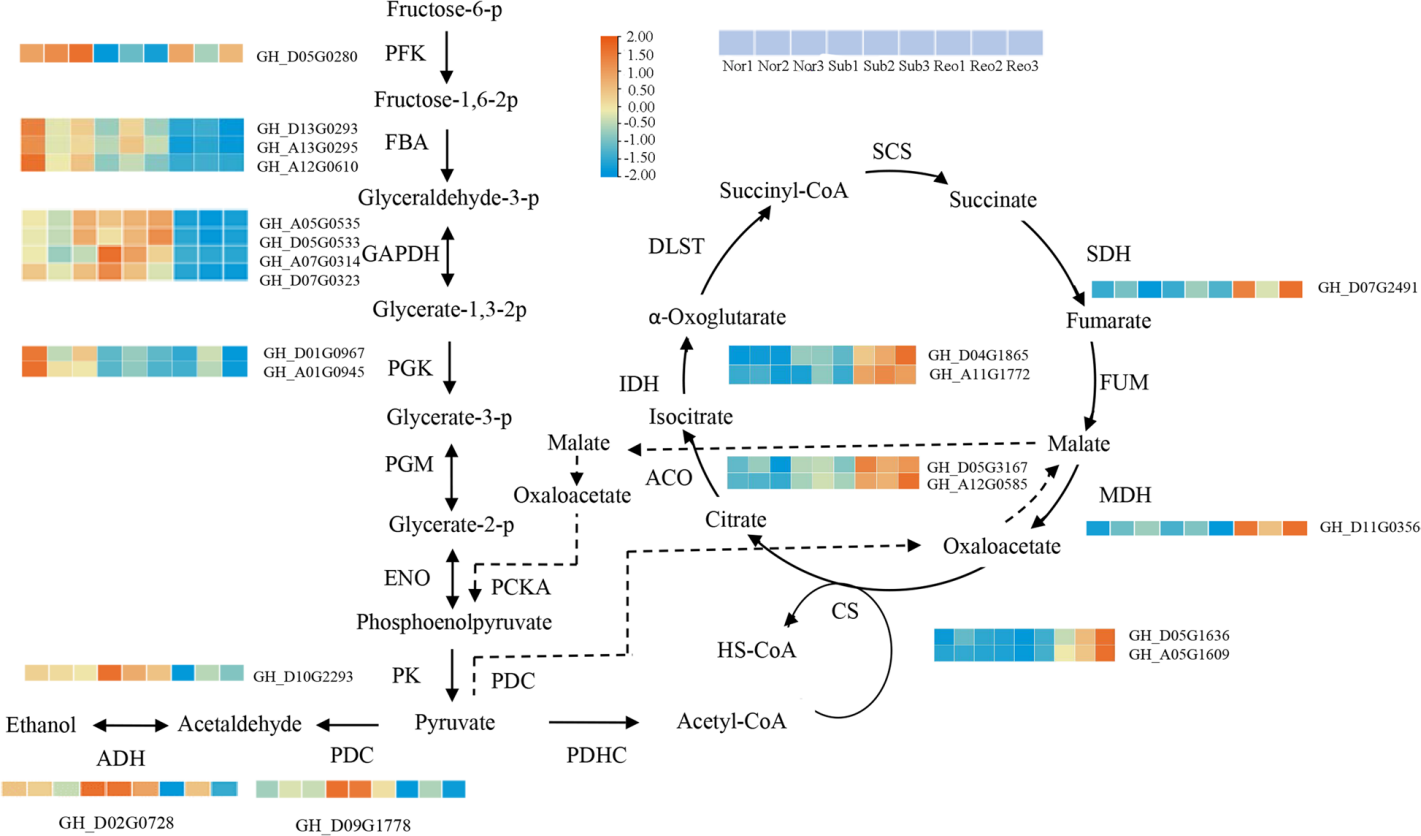
Heatmap of DEGs of the respiratory metabolism pathway. Nor: normal growth; Sub: submergence; Reo: reoxygenation after submergence

Respiratory metabolism not only provides energy for life activities but also provides raw materials for the synthesis of secondary metabolites. To further study the response of respiratory metabolism to submergence stress, DEGs of the glycolytic pathway and the citric acid cycle (EMP-TCA) were further studied through the KEGG pathway (Fig. 8). In the glycolysis pathway, we found that *ADH* and *PDC* were significantly upregulated in the submergence treatment but significantly downregulated in the reoxygenation treatment. *ADH* and *PDC* play important roles in the response to submergence stress. In the submergence treatment, *6-phosphofructokinase (PFK)* and *phosphoglycerate kinase (PGK)* were significantly downregulated. At the same time, we found that the *sucrose synthase genes (SUS)* (GH_A06G0873 and GH_D06G0851) (Fig. S2) were significantly downregulated. ZNL2067 may reduce the rate of carbohydrate decomposition by reducing the expression of *PFK*, *PGK*, and *SUS* to prolong the survival time under submergence stress. Interestingly, there were no significant differences in the expression levels of *citrate synthase (CS), aconitate hydratase (ACO)* and other TCA-related genes between submergence and control treatment. We speculated that the impact of submergence stress on the TCA cycle was not obvious, and its specific mechanism needs to be further verified.

### DEG analysis of the terpene metabolism pathway

**Fig. 9:**
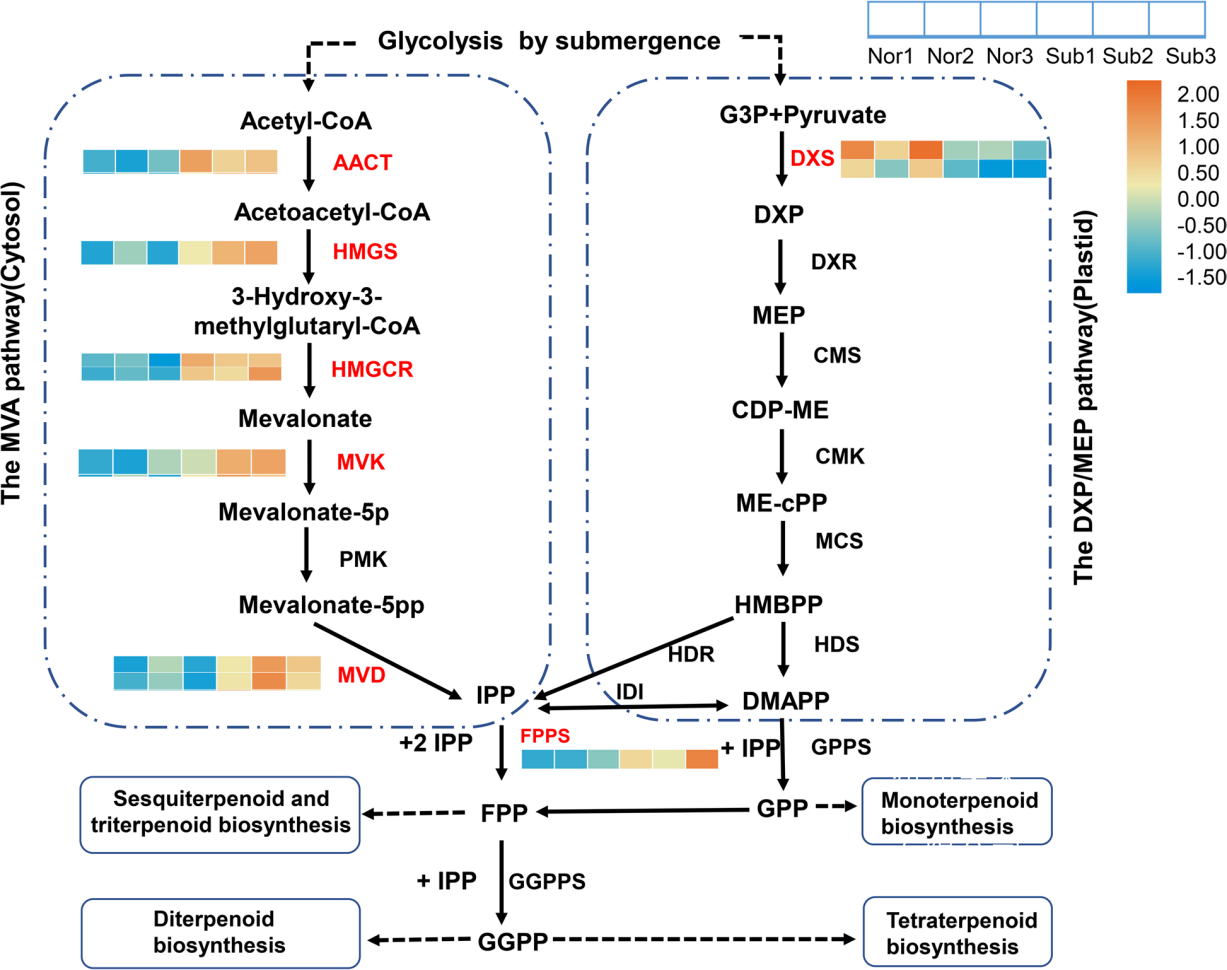
Analysis of the terpenoid biosynthesis pathway and heatmap of DEGs related to monoterpenes, diterpenoids and sesquiterpenes. Nor: normal growth; Sub: submergence

We analyzed the terpene metabolism-related KEGG pathway in response to submergence stress. The KEGG pathways enriched by DEGs mainly included terpenoid backbone biosynthesis (ko00900), monoterpene biosynthesis (ko00902), diterpene biosynthesis (ko00904), sesquiterpenoid and triterpenoid biosynthesis (ko00909), and carotenoid biosynthesis (ko00906). Studies have found that the accumulation of some secondary metabolites enhances the ability to eliminate ROS [31–33]. We further analyzed the terpenoid biosynthetic pathway and found that secondary metabolism-related genes were significantly enriched (Table 3, Fig. 9). ZNL2067 mainly responded to submergence stress through the MVA pathway, not the DXP pathway. In the MVA pathway, there was no downregulation of DEGs.

**Table 3:** DEGs in the terpenoid metabolic pathways under submergence treatment

KEGG Pathway	Gene ID	Description	Log_2_FC(Sub)
Terpenoid backbone biosynthesis	GH_D06G2411	Acetyl-CoA acetyltransferase, AACT	0.68
	GH_A12G2448	Hydroxymethylglutaryl-CoA synthase, HMGS	0.70
	GH_D02G2102	Hydroxymethylglutaryl-coenzyme A reductase, HMGCR	0.71
	GH_D02G2632	Hydroxymethylglutaryl-coenzyme A reductase, HMGCR	1.53
	GH_A11G3372	Mevalonate kinase, MVK	0.82
	GH_D13G0113	Diphosphomevalonate decarboxylase, MVD2	0.70
	GH_A13G0114	Diphosphomevalonate decarboxylase, MVD2	0.81
	GH_A13G0921	Isopentenyl phosphate kinase, IPK	0.61
	GH_D01G0916	Farnesyl pyrophosphate synthase 2, FPPS2	0.80
	GH_D08G1041	1-Deoxy-D-xylulose-5-phosphate synthase, DXS	-0.59
	GH_A08G0948	1-Deoxy-D-xylulose-5-phosphate synthase, DXS	-0.62

### Identification of transcription factors (TFs)

**Fig. 10:**
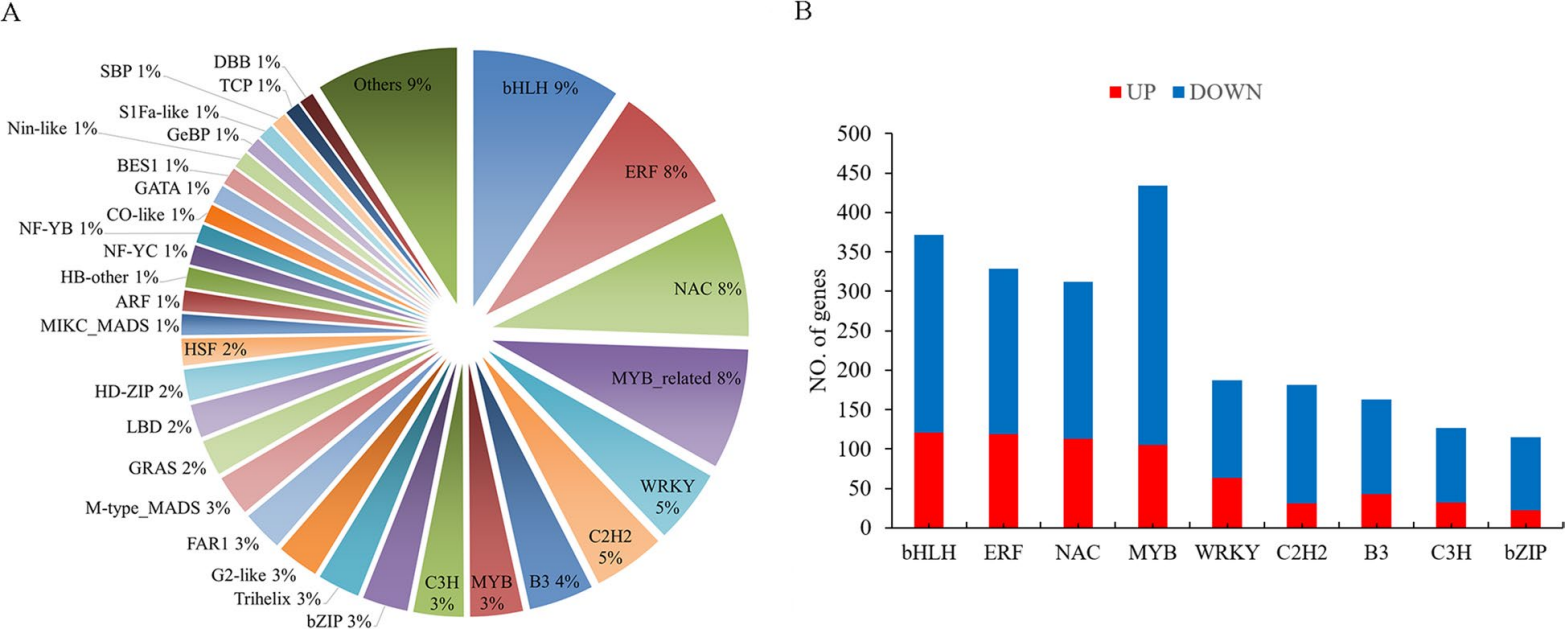
DEGs associated with TF activity in response to submergence stress. **A** The number of enriched TF families. **B** The number of DEGs in different transcription factor families

**Fig. 11:**
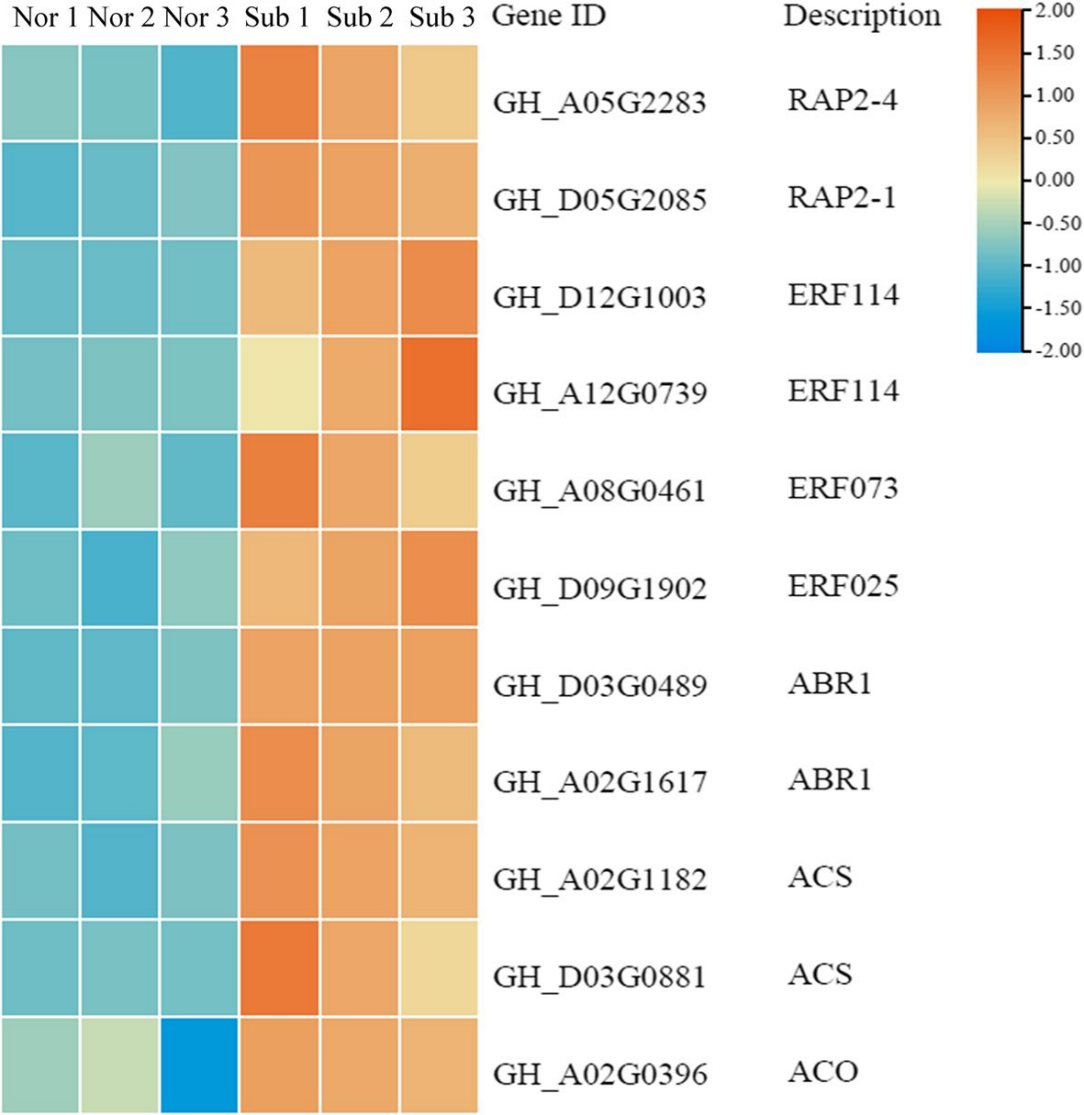
Heatmap analysis of DEGs related to ethylene response and biosynthesis. Nor: normal growth; Sub: submergence

TFs play a vital role in plant resistance to adversity stress. Many TFs were differentially expressed under submergence, and most TFs belonged to the bHLH, ERF, NAC, MYB, WRKY, C2H2, B3, bZIP and C3H families (Fig. 10A). A total of 3,958 TFs were specifically expressed under submergence. Among them, there were 1,150 upregulated genes and 2,808 downregulated genes (Fig. 10B). In the bHLH, ERF, NAC, and MYB families, 121, 119, 113, and 105 genes were upregulated, and 251, 210, 199, and 329 genes were downregulated. The ethylene transcription factors *RAP2-4, RAP2-1, ERF114, ERF073, ERF025* and *ABR1* were significantly upregulated under flooding stress (Fig. 11). These TFs may play important roles in the response to submergence stress.

### Ethylene response and biosynthesis

Plants adjust their response to submergence by altering the balance between phytohormone synthesis and transportation [34]. The rapid synthesis of ethylene is one of the important ways for plants to actively cope with waterlogging or submergence stress [22, 35–39]. In this study, 8 DEGs related to ethylene signaling and 3 ethylene synthesis genes were identified (Fig. 11). The *ACO* and *ACS* genes were significantly upregulated under submergence and reoxygenation treatment (Fig. 11), indicating that the *ACO* and *ACS* genes had stronger ethylene synthesis activity under submergence and reoxygenation conditions.

### Verification of RNA-seq data

**Fig. 12:**
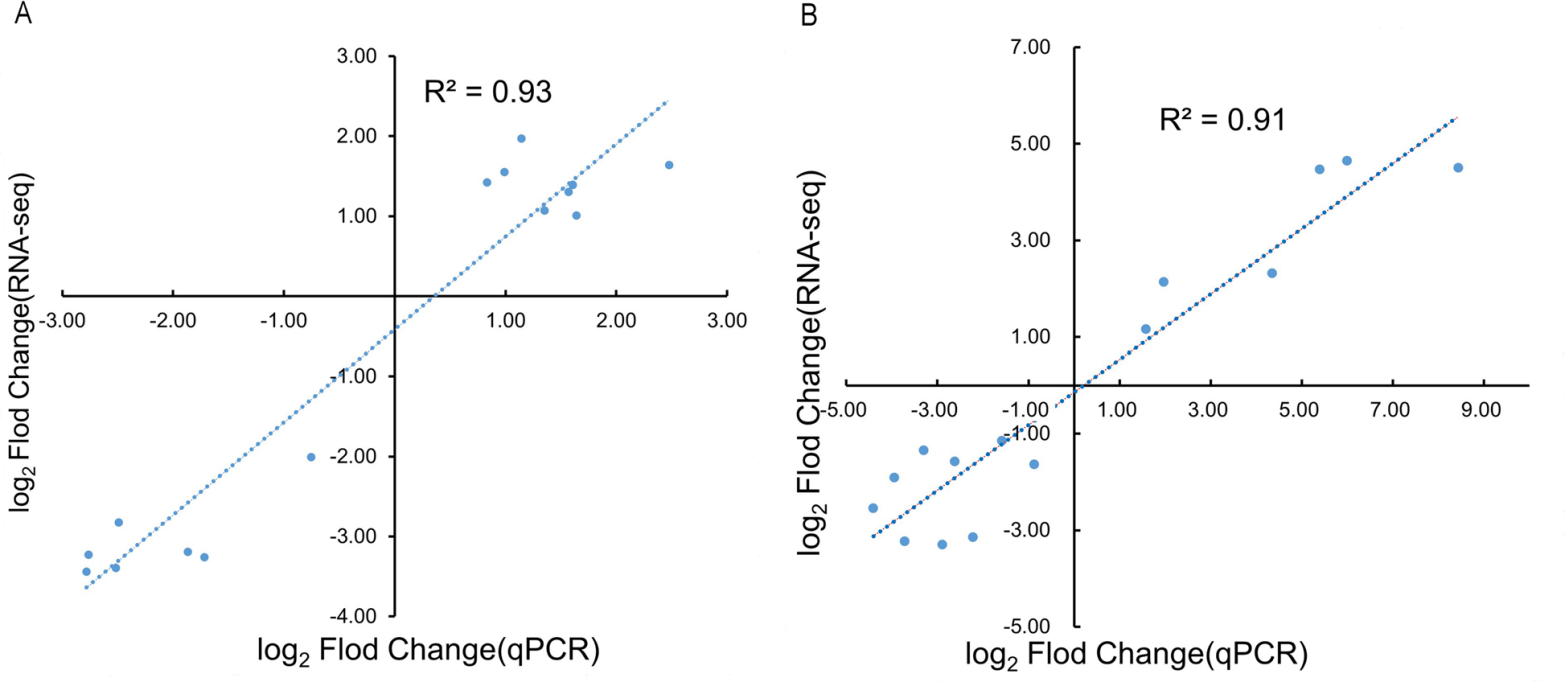
Correlation analysis between RT-qPCR and RNA-seq data. **A** Submergence (Sub); **B** Reoxygenation after submergence (Reo)

To assess the reliability of the transcriptome data, we performed RT-qPCR using the same RNA-seq samples. Thirty DEGs were selected for RT-qPCR validation, including 14 upregulated and 16 downregulated genes (Table S1). The results of RT-qPCR for the 30 DEGs were consistent with the RNA-seq data (Fig. S3), indicating that the two sets of RNA-seq data were reliable (Fig. 12).

## Discussion

With the current climate change, flooding events are becoming challenging problems, with severe impacts on global ecosystems [40, 41]. Therefore, research on the mechanism of plant water tolerance has always been an important agricultural issue. In this study, RNA-seq technology was used to reveal the transcriptome changes in cotton in response to submergence. We studied the morphological changes in ZNL2067 and ZL100 after submergence and reoxygenation (Fig. 1). Physiological and biochemical measurements revealed that ZNL2067 withstood short-term submergence stress (Fig. 2). Therefore, ZNL2067 was chosen as the research material to examine the mechanism of submergence stress.

### Respiratory adaptation

Energy deficiency and respiratory depression caused by hypoxia are some of the most serious problems faced by flooded plants. Previous studies have shown that plants obtain the necessary energy supply to cope with energy shortages caused by flooding by accelerating glycolysis and ethanol fermentation [42]. As the submergence time increases, the accumulation of anaerobic metabolites eventually leads to plant death [43]. RAN-seq data showed that the expression of 6-phosphofructase kinase (*PFK, GH_D05G0280*), phosphoglycerate kinase (*PGK, GH_A01G0945* and *GH_D01G0967*) and sucrose synthase genes (*SUS*, *GH_A06G0873* and *GH_D06G0851*) was significantly down-regulated. This may indicate that ZNL2067 can delay the rate of carbohydrate breakdown by reducing the transcriptional expression levels of *PFK*, *PGK* and *SUS* genes. At the same time, alcohol dehydrogenase (*ADH*, *GH_A02G0728*) and pyruvate decarboxylase (*PDC*, *GH_D09G1778*) were significantly upregulated expressed. TCA is a ubiquitous metabolic pathway in aerobic organisms that is distributed in mitochondria [44–46]. There was no significant difference in the genes involved in the citric acid cycle (TCA) under submergence and normal growth. This indicated that TCA can still maintain basic energy metabolism after submergence for 3 days, which may be related to the formation of ARs and the removal of active oxygen.

### ROS scavenging through the terpene biosynthetic pathway

Secondary metabolites are closely related to respiratory metabolism. Many intermediate products in the process of respiration are used as raw materials for plants to synthesize secondary metabolites, and pyruvate, GA-3P and acetyl-CoA are important upstream raw materials for the synthesis of the terpene biosynthesis pathway [47]. Transcriptome data indicated that the expression of pyruvate, glyceraldehyde 3-phosphate and acetyl-CoA genes was changed by submergence stress, which in turn regulated the metabolic pathways of terpenoids. Hypoxia under submergence stress increases plant ROS levels [33, 48, 49]. The accumulation of ROS results in serious damage, such as lipid peroxidation, protein oxidative damage and DNA oxidative damage, to plant cells [50, 51]. We found that MDA values increased significantly after 3 days of submergence stress and decreased significantly after reoxygenation (Fig. 2C). Plants eliminate ROS accumulation mainly through secondary metabolites and antioxidant enzyme systems [14, 33]. We discovered the KEGG pathways of some secondary metabolites, including sesquiterpenoid and triterpenoid biosynthesis, terpenoid backbone biosynthesis, monoterpenoid biosynthesis, and diterpenoid biosynthesis. In the terpenoid backbone biosynthesis pathway, the mevalonate pathway that occurred in the cytoplasm was the main pathway employed in response to submergence stress. DXS is an important rate-limiting enzyme in the pyruvate/glyceraldehyde phosphate pathway. Its expression was significantly downregulated under submergence stress. We further studied the changes in key genes in the terpene metabolism pathway, such as *TPS, 2-OGD, and SM*. The expressions of these three genes were significantly up-regulated under submergence stress (Table S2). *TPS* is a key gene in the synthesis of terpene compounds [52]. *SM* is a gene that regulates cholesterol synthesis [53]. Carotene and lycopene are tetraterpenes, sterols and squalene are triterpenes, and these tetraterpene compounds have strong antioxidant properties [54, 55]. The results showed that there was no significant difference in POD activity among the treatments (Fig. 2D). This is consistent with the results of previous studies [56]. We speculated that ZNL2067 may have eliminated excess ROS after being submerged for 3 days. Therefore, we hypothesized that genes related to terpenoid metabolic pathway may play an important role in ROS scavenging under submergence stress.

### Specific expression of TFs

TF families, such as bHLH, ERF, NAC, MYB, WRKY and bZIP, were significantly differentially expressed under submergence stress. Studies have shown that overexpressing *bHLH4* and *bHLH6* increased the accumulation of Phaenopsis volatile monoterpenes [57]. The expression level of *AaERF1/2* increased under the induction of jasmonic acid and promoted the accumulation of artemisinin in *Artemisia annua* [58]. Artemisia annua *NAC1* binds to the *ADS* promoter and upregulates its expression to increase the artemisinin content and enhance the drought stress tolerance of plants [59]. Soybean *MYBZ2* is an inhibitor of vinblastine synthesis [60]. Overexpression of *CrWRKY1* significantly promoted the synthesis of indole alkaloids in Catharanthus roseus [61]. Artemisia annua *AaAPK1* phosphorylates *bZIP1* to achieve posttranscriptional regulation of artemisinin synthesis [62]. In the absence of oxygen, plant cysteine oxidase (PCO) cannot destroy the ERF-VII transcription factor, leading to the activation of hypoxia response gene (HRG) transcription [63]. Five ERF-VII genes (*HRE1, HRE2, RAP2.2, RAP2.3,* and *RAP2.12*) are considered to be the key regulators of hypoxia tolerance in *Arabidopsis* [64, 65]. The gene *ZmEREB180* positively regulates the growth and development of ARs and the level of ROS in maize (Yu, et al. 2019). We found that the ethylene transcription factor *RAP2* was significantly upregulated under submergence stress. This may be one of the reasons why ZNL2067 was resistant to submergence.

## Conclusions

**Fig. 13:**
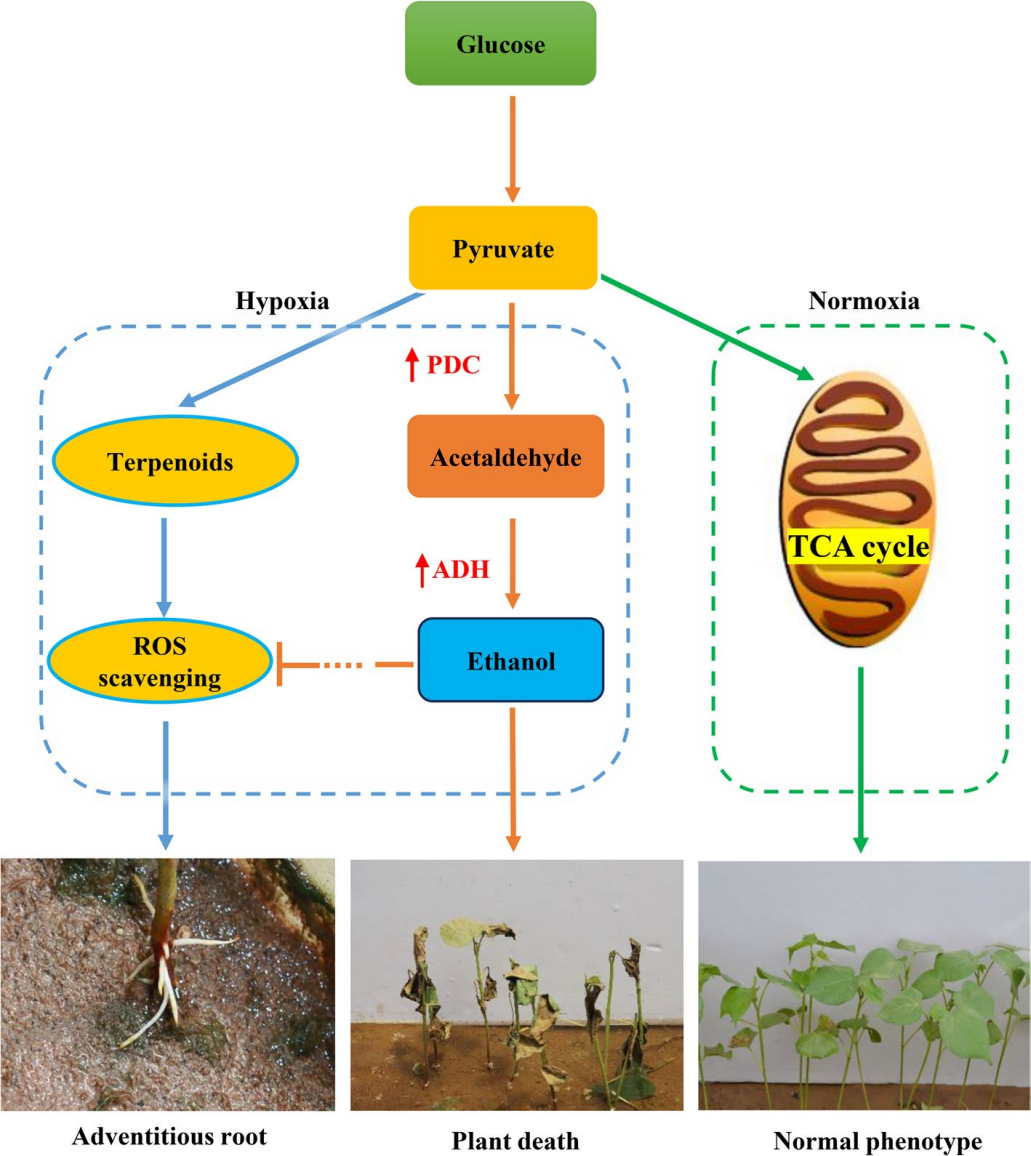
Hypothetical model of cotton responding to submergence stress. Line with blocked end indicates inhibitory effects, and arrow indicates positive stimuli. Under normoxia, pyruvate is completely oxidized to produce ATP through the TCA cycle to maintain the normal growth and development of cotton. Under hypoxia condition, cotton can obtain the energy needed for temporary survival by increasing the transcriptional expression levels of *ADH* and *PDC* genes; the genes related to the regulation of terpenoid biosynthesis pathway were up-regulated possibly to eliminate ROS and improve the tolerance of cotton to submergence stress

The physiological and transcriptional changes in cotton under submergence and post-submergence reoxygenation stress were investigated to explore the response mechanism of cotton to flooding stress. Under normoxia, pyruvate is completely oxidized to produce ATP through the TCA cycle to maintain the normal growth and development of plants [66, 67]. Based on the results of RNA-seq analysis, cotton could slow down the rate of carbohydrate degradation and obtain the energy required for survival by reducing the transcription levels of *SUS*, *PGK* and *PFK* genes and increasing the transcriptional expression levels of *ADH* and *PDC* genes under hypoxic conditions. At the same time, the genes related to the regulation of terpenoid biosynthesis pathway were up-regulated possibly to eliminate ROS and improve the tolerance of cotton to submergence stress. Based on these results, we derived a hypothetical model of the cotton response to submergence (Fig. 13).

## Material and methods

### Materials and design

The experiment was conducted in a modern solar greenhouse (N: 39°10 ', E: 114°35 ') at the Institute of Cotton Research of the Chinese Academy of Agricultural Sciences (ICR, CAAS) in 2020. The submergence identification pools (2 m × 20 m) in modern solar greenhouses are separated by concrete walls to prevent the soil moisture in the different pools from infiltrating each other. Three treatments were designed: complete submergence (3d), reoxygenation after submergence (3d) and normal growth. In the submergence treatment, the entire plant was completely submerged during the cotton three-leaf period. In the control treatment, water was well maintained (the soil moisture was 60–70%). Each treatment had three independent biological replicates.

The experimental materials were planted in single rows with a length of 2 m and a spacing of 20 cm. When the soil temperature was above 15 °C, the seeds were evenly planted in the identification pool and watered well. After 7 days, weak and deformed seedlings were removed, and 20–30 cotton seedlings with uniform growth and robust growth were retained in each plot. When the seedlings reached three true leaves, we submerged them all in water, keeping the water level 1–3 cm higher than the seedlings. When the average water temperature was 25℃ and the average temperature was 36℃, the time of submergence treatment for 3 days. Water was then drained and growth was normal for 3d.

### Observation of the internal structure of leaf tissue

Paraffin sections were used to observe the changes in leaf tissue in ZNL2067 and ZL100 from three treatments. After stress treatment, the leaves were quickly cut into 5 mm × 10 mm pieces, and then fixed with FAA fixing solution. The slices were made by paraffin sectioning method and sliced with a microtome (RM2016) with a thickness of 5 μm. Then stained with safranine and solid green, and sealed with neutral gum. Finally, a light microscope (NIKON ECLIPSE E100) was used to observe and take photos. We used CaseViewer software to observe longitudinal Sects. (200 ×) of paraffin sections of leaves. Palisade tissue thickness, spongy tissue thickness, and leaf thickness were measured, and palisade tissue/spongy tissue ratio, CTR and SR were calculated. Three replicates were measured for each treatment, and 5 readings were taken for each replicate.

### Dry matter determination

10 representative cotton plants were randomly selected from three treatments, separated by roots, stems, and leaves, placed in an oven. First, they were dried at 105℃ for 30 min and then at a temperature of 75 ℃ to a constant weight. Finally, the dry matter weight was calculated.

### Net photosynthetic rate determination

The photosynthetic parameters of the main stem functional leaves of ZNL2067 were measured by a Li-6400 portable photosynthesis meter (produced by LI-COR, USA) on the day of submergence for 3 days and reoxygenation for 3 days. Three replicates were measured for each treatment, and 10 readings were taken for each replicate.

### MDA and POD measurements

0.1 g of leaf tissue was weighed in three treatments, and 1 mL of extract was added to homogenize in an ice bath. After centrifugation at 8000 g for 10 min at 4 ℃, the supernatant was removed and placed on ice with three biological replicates for each sample. Samples were taken to determine the activity of POD and MDA, and a POD assay kit and MDA assay kit (#A084-3–1, #A003-3–1, Jiancheng Bioengineering Institute, Nanjing, Jiangsu, China) were used to measure the enzyme activity.

### cDNA library construction and sequencing

Representative samples of ZNL2067 were randomly selected from the Nor, Sub and Reo treatments. RNA was separately extracted from roots, stems, and leaves, and then, equal amounts of RNA from roots, stems, and leaves were mixed. Each treatment was repeated three times independently.

We isolated and purified total RNA using TRIzol (Invitrogen, Carlsbad, CA, USA). RNA was quantified using NanoDrop ND-1000 (NanoDrop, Wilmington, DE, USA), and the RNA integrity was assessed using a Bioanalyzer 2100 (Agilent, CA, USA) with a RIN of > 7.0. The cDNA library was built utilizing the methods of Fan et al*.* [68]. The average insert length in the cDNA library was 300 ± 50 bp. Finally, we performed 2 × 150 bp paired-end sequencing (PE150) on an Illumina NovaSeq™ 6000 (LC-Bio Technology CO., Ltd., Hangzhou, China).

### Identification of differentially expressed genes (DEGs)

We used StringTie (version: stringtie-1.3.4d.Linux_x86_64) to assemble the mapped reads for each sample [69]. All transcriptomes from the samples were merged to reconstruct a comprehensive transcriptome using the gffcompare software (version: gffcompare-0.9.8.Linux_x86_ 64). We estimated the expression levels of all transcripts using StringTie and Ballgown and determined mRNA expression levels by calculating FPKM values. The differentially expressed mRNAs and genes were selected with log_2_fold change (FC) > 1 or log_2_ (FC) < -1 and *p* value < 0.05 by R package edge R [70]. We used TBtools software to display heatmaps [71].

### Gene Ontology (GO) and KEGG pathway enrichment analyses

We performed GO enrichment analysis of DEGs using the GOseq R package [72], and the length bias of DEGs was corrected. GO terms (*p* value < 0.05) were considered significantly enriched by DEGs. KOBAS77 software was used to test the statistical enrichment of DEGs in KEGG pathways. All DEGs were compared against the GO and KEGG [73–75].

### Real-time quantitative PCR (RT-qPCR) validation and analysis

We selected thirty DEGs to validate the reliability of the transcriptome database. Thirty pairs of primers were designed using the Primer 6.0 software (Table S1), and RT-qPCR was performed [68]. The *Actin* gene was used as a reference.

### Data Processing

SPPS(Ver.21) and EXCEL software were used for statistical analysis. One-way analysis of variance (ANOVA) or Duncan's method was used to compare the significant levels of differences between different treatments (α = 0.05).

## Supplementary Information

**Additional file 1.**

**Additional file 2.**

**Additional file 3.**

**Additional file 4.**

## Data Availability

Data generated by RNA sequencing were deposited in the NCBI repository [Accession number: PRJNA629890]. ZNL2067 and ZL100 were from the laboratory of Wuwei Ye, Institute of Cotton Research of Chinese Academy of Agricultural Sciences.

## Abbreviations

DEGs: Differentially expressed genes
GO: Gene Ontology
KEGG: Kyoto Encyclopedia of Genes and Genomes
LDH: Lactate dehydrogenase
PDC: Pyruvate decarboxylase
ADH: Alcohol dehydrogenase
ROS: Reactive oxygen species
MVA pathway: Mevalonate pathway
DXP: Pyruvate/glyceraldehyde-3-phosphate
GA-3P: Glyceraldehyde 3-phosphate
EMP-TCA: Citric acid cycle
PFK: 6-Phosphofructokinase
PGK: Phosphoglycerate kinase
SUS: Sucrose synthase genes
CS: Citrate synthase
ACO: Aconitate hydratase
LCYB: Lycopene β-cyclase
TPS: Terpene synthase
2-OGD: 2-Oxoglutarate-dependent dioxygenase
SM: Squalene monooxygenase
PCD: Programmed cell death
PCO: Plant cysteine oxidase
AACT: Acetyl-CoA acetyltransferase
HMGS: Hydroxymethylglutaryl-CoA
HMGCR: Hydroxymethylglutaryl-CoA reductase synthase
MVK: Mevalonate kinase
PMK: Phosphomevalonate kinase
MVD: Mevalonate diphosphate decarboxylase
IPP: Isopentenyl pyrophosphate
DXS: 1-Deoxy-D-xylulose-5-phosphate synthase
DXR: 1-Deoxy-D-xylulose-5-phosphate reductoisomerase
CMS: 2-C-methyl-D-erythritol 4-phosphate cytidylyltransferase
CMK: 4-Diphosphocytidyl-2-C-methyl-D-erythritol kinase
MCS: 2-C-methyl-D-erythritol 2,4-cyclodiphosphate synthase
HDS: 4-Hydroxy-3-methylbut-2-enyl diphosphate synthase
GPPS: Geranyl diphosphate synthase
FPPS: Farnesyl diphosphate synthase
GGPPS: Geranylgeranyl diphosphate synthase
