# Supplementary material for: Changes in terpene biosynthesis and submergence tolerance in cotton
Source: BMC Plant Biol. 2023 Jun 21;23:330. doi: 10.1186/s12870-023-04334-4 (PMC10283293; doi:10.1186/s12870-023-04334-4)
Supplement: Supplementary file 1 — Additional file 1. [file 12870_2023_4334_MOESM1_ESM.docx]

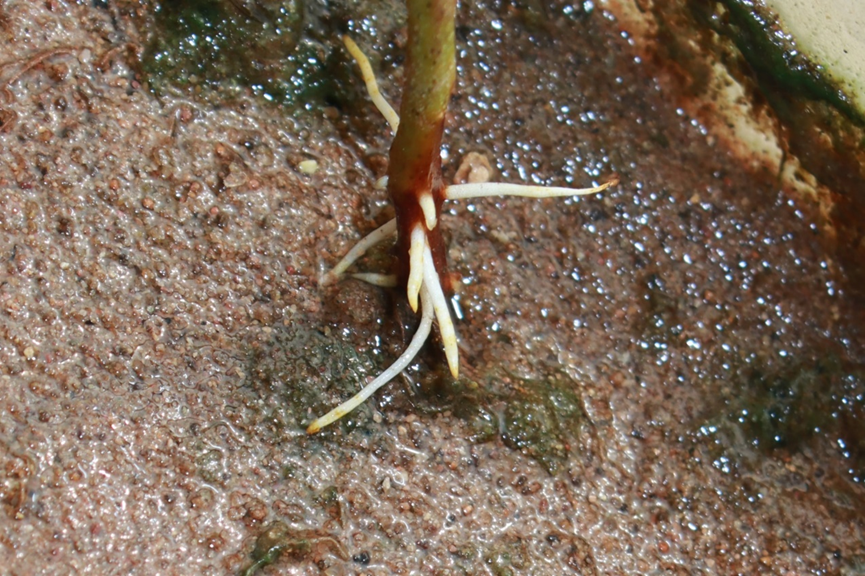


**Fig. S1** ARs formation of ZNL2067 after being submerged for 3 days.


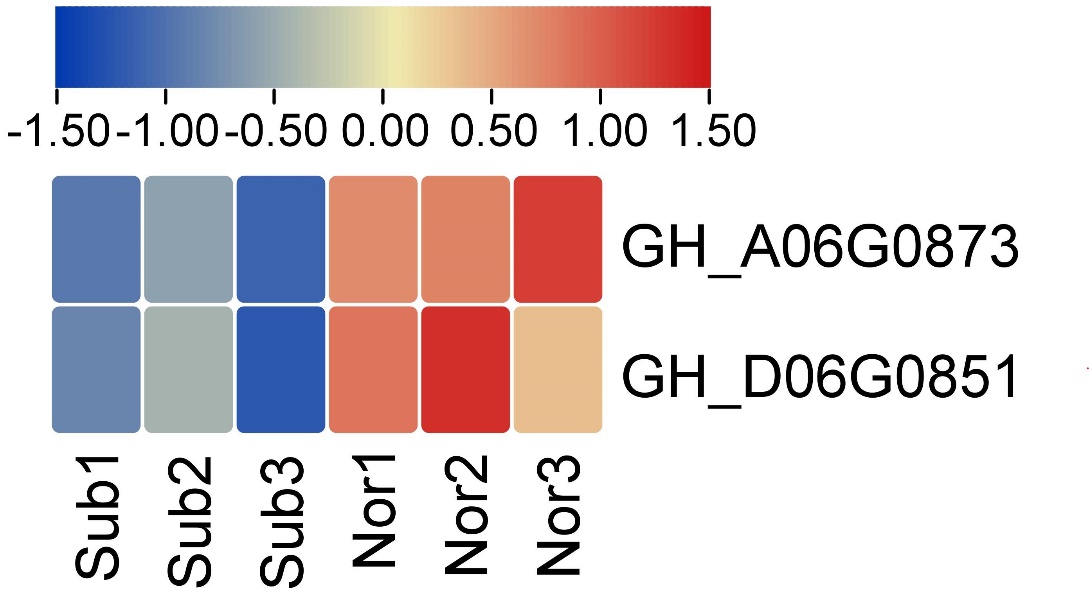


**Fig. S2** Heat map of sucrose synthase. Red and blue represent high and low expression levels, respectively (FPKM).


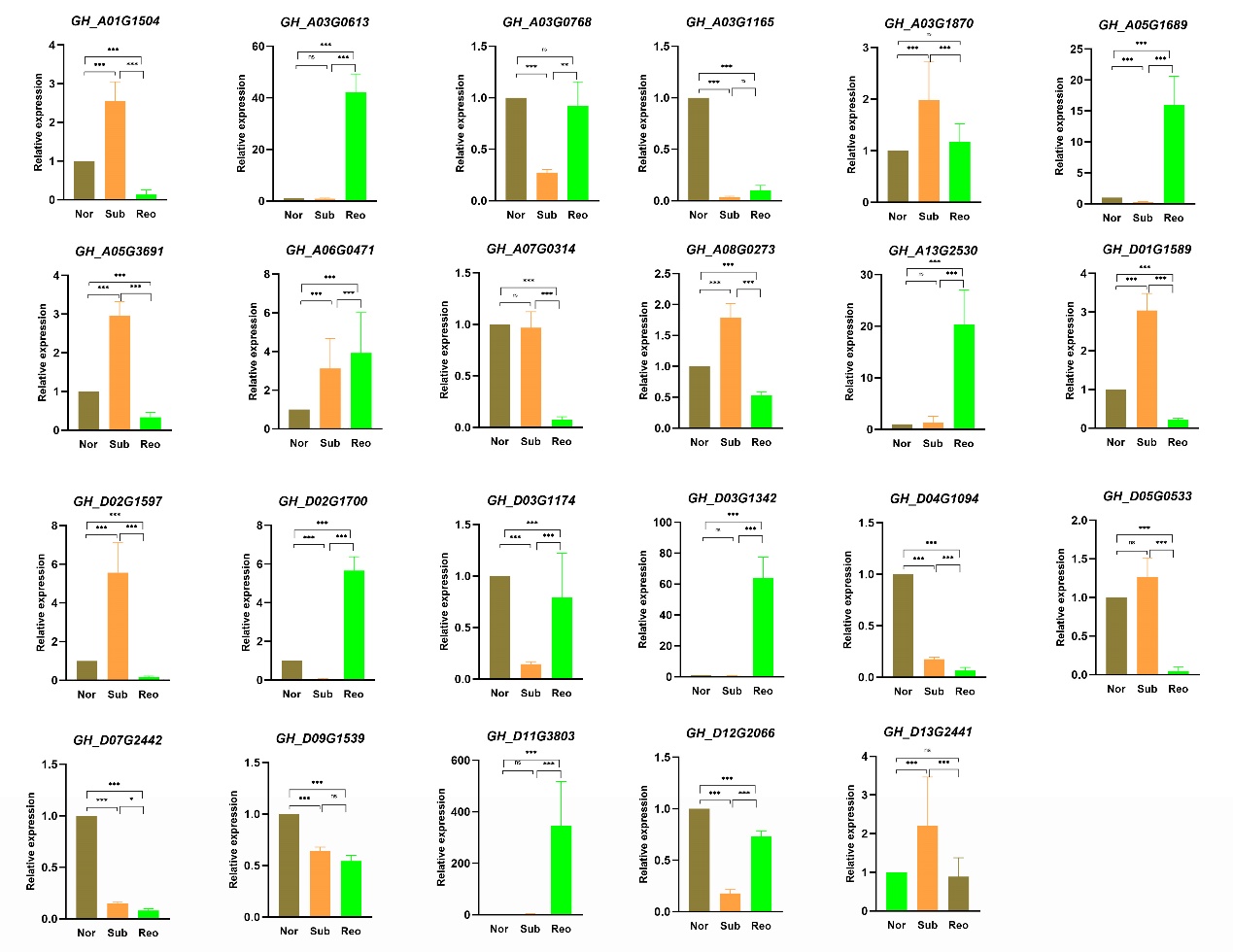


**Fig. S3** Expression of 23 DEGs. Error bars are the standard deviation (SD) of three biological replicates in each treatment group. *: P < 0.05, **: P < 0.01, ***: P < 0.001.
