# Supplementary material for: Changes in terpene biosynthesis and submergence tolerance in cotton
Source: BMC Plant Biol. 2023 Jun 21;23:330. doi: 10.1186/s12870-023-04334-4 (PMC10283293; doi:10.1186/s12870-023-04334-4)
Supplement: Supplementary file 2 — Additional file 2. [file 12870_2023_4334_MOESM2_ESM.docx]

Table S1 Primers pairs for qRT-PCR

| **Gene ID** | **Function annotation** | **Primers(5'-3')** |
| --- | --- | --- |
| GH_D13G2441  GH_D02G1597  GH_A03G1870  GH_A08G0273  GH_D01G1589  GH_A05G3691  GH_A01G1504  GH_A06G0471  GH_D03G1342  GH_D12G2066  GH_A03G0768  GH_D07G2442  GH_A05G1689  GH_D04G1094  GH_D03G1174  GH_A07G0314  GH_D05G0533  GH_D09G1539  GH_A03G1165  GH_D02G1700  GH_A13G2530  GH_A03G0613  GH_D11G3803  Actin | Hexokinase-3  Probable ribose-5-phosphate isomerase 2  Cyclin-U1-1  Xyloglucan endotransglucosylase/hydrolase protein 22  UDP-glycosyltransferase 74B1  Very-long-chain enoyl-CoA reductase  UDP-glycosyltransferase 74B1  Gamma-glutamylcyclotransferase 2-3  Phosphomethylpyrimidine synthase  Probable pectate lyase 5  Probable pectate lyase 5  Carbonic anhydrase  Thiamine thiazole synthase 2  Alpha carbonic anhydrase 1  Probable pectate lyase 5  NADP-dependent glyceraldehyde-3-phosphate dehydrogenase  NADP-dependent glyceraldehyde-3-phosphate dehydrogenase  Pyruvate kinase 1, cytosolic  Sphinganine C4-monooxygenase 2  Probable pectate lyase 5  Alpha-glucan phosphorylase, H isozyme  Phosphomethylpyrimidine synthase  Senescence-specific cysteine protease SAG39 | F: CTGGCATCGTTGGGATCTTG  R: ACGTGCCGTGCAATATCTTC  F: CCAAGCTGAGGAAAGACTG  R: TCCAAGCTCACCTGCAACTA  F: GATCCAATCCAAGCCGACAC  R: CCCTGACGCCATGAAATACG  F: CTCGAGGTGGCGTTGTTAAG  R: AGGACCAGACACATGCATCA  F: TGCTCGTGCCTTAGAGGAAA  R: AGTTGGTTGCACCATGTCAC  F: TCACGCAGTGGAAGAGAGTT  R: TTTGATCCCGGAGGTACTGG  F: CAGTTCAAAGGGCACGTGAT  R: AGCCACCTTCATCGAATCCA  F: CTTTGCTGTGGGAGTTCTCG  R: GCGTCCCTCTGTGATCAGTA  F: ACTGCCATGTCCTTCCATGA  R: CAGCCAGGAATTCAGCACTC  F: GCCATTGGAGGAAGTGCAAA  R: AATGCACCGTTCACCATCAG  F: TGCCATTGGAGGAAGTGCTA  R: GGTCACCTTCCGATCTCCAA  F: AGAGCAAGTGACAGCTGAGT  R: CCTTGGCAAGCTCACCATAC  F: CAAACTGGGCTTTGGTGTCA  R: ACCTTGGACTCCATCACGTT  F: GCAGCTGAGCTTCATTTGGT  R: GTGGATCAGCGTTGCTTTCT  F: TGCCATTGGAGGAAGTGCTA  R: GCACCGTTCACCATTAGGTC  F: CACTTCCAAGATCCCGCTTG  R: GGAAAGCCAGCCAAGTGAAA  F: TCACCATGCATCCTGGAGTT  R: CGGCATCCTCGAGTACGATA  F: TCCCACCTGAGAAGGTGTTC  R: CTGTCCACAACACGAGTGAC  F: CACCATGGGCGGAACATTAG  R: GTTTCCGGGAAGCTTCATCC  F: CATGCAAGTCACCATTGCCT  R: TTGTAGGATCAGCACTGCCA  F: ATATGGCCCACCAAGTTCCA  R: CGAAGACCAGAAAGCAGGT  F: ACTGCCATGTCCTTCCATGA  R: CAGCCAGGAATTCAGCACTC  F: GGTCTCATTGTCCGAGCAAC  R: GCTGATATGGGCAACTTGGG  F: ATCCTCCGTCTTGACCTTG  R: TGTCCGTCAGGCAACTCAT |
