# Supplementary material for: Changes in terpene biosynthesis and submergence tolerance in cotton
Source: BMC Plant Biol. 2023 Jun 21;23:330. doi: 10.1186/s12870-023-04334-4 (PMC10283293; doi:10.1186/s12870-023-04334-4)
Supplement: Supplementary file 3 — Additional file 3. [file 12870_2023_4334_MOESM3_ESM.docx]

Table S2 Changes of expression levels of *TPS*, *2-OGD* and *SM* in the terpenoid metabolic pathways under submergence treatment

| KEGG Pathway | Gene ID | Description | Log_2_FC |
| --- | --- | --- | --- |
| Monoterpenoid biosynthesis | *GH_D05G2948* | Terpene synthase, TPS | 5.66 |
| Diterpenoid biosynthesis | *GH_A07G2040*  *GH_A13G2327*  *GH_A13G2332*  *GH_D13G2323*  *GH_D13G2329* | 2-oxoglutarate-dependent dioxygenase, 2-OGD  2-oxoglutarate-dependent dioxygenase, 2-OGD  2-oxoglutarate-dependent dioxygenase, 2-OGD  2-oxoglutarate-dependent dioxygenase, 2-OGD  2-oxoglutarate-dependent dioxygenase, 2-OGD | 1.27  3.93  1.44  3.02  1.15 |
| Sesquiterpenoid and triterpenoid biosynthesis | *GH_scaffold7085_objG0001* | Squalene monooxygenase, SM | 3.23 |
