# Supplementary material for: Changes in terpene biosynthesis and submergence tolerance in cotton
Source: BMC Plant Biol. 2023 Jun 21;23:330. doi: 10.1186/s12870-023-04334-4 (PMC10283293; doi:10.1186/s12870-023-04334-4)
Supplement: Supplementary file 4 — Additional file 4. [file 12870_2023_4334_MOESM4_ESM.docx]

Table S3 The expression of *Actin* gene

| Tissues | CK (CT) | Submergence (CT) | Reoxygenation (CT) |
| --- | --- | --- | --- |
| Root | 22.125  22.065  22.095 | 22.812  22.693  22.752 | 23.091  23.087  23.089 |
| Stem | 22.207  22.248  22.227 | 22.881  22.917  22.899 | 23.108  23.228  23.168 |
| Leaf | 22.240  22.461  22.350 | 22.854  23.050  22.952 | 22.183  24.203  23.193 |
